# What Are You Feeling? Using Functional Magnetic Resonance Imaging to Assess the Modulation of Sensory and Affective Responses during Empathy for Pain

**DOI:** 10.1371/journal.pone.0001292

**Published:** 2007-12-12

**Authors:** Claus Lamm, Howard C. Nusbaum, Andrew N. Meltzoff, Jean Decety

**Affiliations:** 1 Department of Psychology and Center for Cognitive and Social Neuroscience, The University of Chicago, Chicago, Illinois, United States of America; 2 Institute for Learning and Brain Sciences, University of Washington, Seattle, Washington, United States of America; Lund University, Sweden

## Abstract

**Background:**

Recent neuroscientific evidence suggests that empathy for pain activates similar neural representations as the first-hand experience of pain. However, empathy is not an all-or-none phenomenon but it is strongly malleable by interpersonal, intrapersonal and situational factors. This study investigated how two different top-down mechanisms – attention and cognitive appraisal - affect the perception of pain in others and its neural underpinnings.

**Methodology/Principal Findings:**

We performed one behavioral (N = 23) and two functional magnetic resonance imaging (fMRI) experiments (N = 18). In the first fMRI experiment, participants watched photographs displaying painful needle injections, and were asked to evaluate either the sensory or the affective consequences of these injections. The role of cognitive appraisal was examined in a second fMRI experiment in which participants watched injections that only appeared to be painful as they were performed on an anesthetized hand. Perceiving pain in others activated the affective-motivational and sensory-discriminative aspects of the pain matrix. Activity in the somatosensory areas was specifically enhanced when participants evaluated the sensory consequences of pain. Perceiving non-painful injections into the anesthetized hand also led to signal increase in large parts of the pain matrix, suggesting an automatic affective response to the putatively harmful stimulus. This automatic response was modulated by areas involved in self/other distinction and valence attribution – including the temporo-parietal junction and medial orbitofrontal cortex.

**Conclusions/Significance:**

Our findings elucidate how top-down control mechanisms and automatic bottom-up processes interact to generate and modulate other-oriented responses. They stress the role of cognitive processing in empathy, and shed light on how emotional and bodily awareness enable us to evaluate the sensory and affective states of others.

## Introduction

Recent evidence from functional neuroimaging studies suggests that the perception of pain in others activates similar neural circuits as the first-hand experience of pain - especially in regions processing the affective-motivational dimension of pain, such as the anterior insula and the anterior cingulate cortex [1]–[9]. These findings stress the importance of implicit and automatically shared neural representations between self and other for the experience of empathy [10], [11].

Recent models of empathy, however, also emphasize the role of top-down processes such as perspective taking and self/other awareness [12], [13]. These models emphasize that empathy is not an all-or-none phenomenon. Its experience is malleable by a number of factors including personality traits and the type of situation in which social interaction occurs. However, little is known about the neural mechanisms underlying the modulation of empathy. For example, physiological research has shown that evaluating either the sensory or the affective consequences of first-hand pain recruits neural pathways specifically involved in sensory discrimination and affective-motivational processing [14]. It remains unclear whether this also hold true for the perception of pain in others. We also have only cursory knowledge about how cognitive processes such as deliberate appraisal of the other's situation modulate the empathic reaction to the pain of others.

The aim of the present study was to investigate how two cognitive mechanisms of top-down control – attention and appraisal – affect the psychological and neural correlates of empathic responding. To this end, we performed one behavioral experiment and two subsequent fMRI experiments. The behavioral experiment served for stimulus validation and design optimization, while the fMRI experiments assessed the roles of evaluative focus and cognitive appraisal on brain activity during empathy for pain. More specifically, the first fMRI experiment explored whether focusing on the sensory or the affective consequences of pain in others results in modulation of the hemodynamic signal in areas of the pain matrix processing sensory or affective information. The second fMRI experiment investigated how these responses are modulated by evaluating a putatively harmful situation which is actually not painful. In addition, a number of behavioral and dispositional measures were taken in order to assess the effects of individual differences in empathy and emotion contagion on brain activation during empathizing.

The question whether focusing on the sensory or affective consequences of another's pain recruits distinct neural networks springs from an ongoing controversy about whether only the affective-motivational or also the somatosensory-discriminative components of pain processing are involved in empathy for pain. Most fMRI studies to date suggest that witnessing another's pain does not recruit areas that are typically involved in coding the sensory aspects of one's own pain - such as the somatosensory cortex (SI/SII and posterior insula) for thermal or mechanical pain (e.g., [3], [4], [6], [8]). In contrast, transcranial magnetic stimulation (TMS) [15], [16], electroencephalography [17], [18] and magnetoencephalographic measurements [2] suggest a role of sensorimotor representations during the perception of pain in others. One explanation for these discrepancies between fMRI and other measures is the way in which participants observed the targets. For instance, in the TMS studies participants were explicitly instructed to focus on what the depicted person may have felt during the injection of a needle into the hand or the foot – directing their attention to the sensory aspects of pain, as well as to the affected body part. This interpretation is supported by a positron emission tomography (PET) study showing that focusing on the location of pain on one's own body increased regional cerebral blood flow in the contralateral primary somatosensory cortex and the inferior parietal lobule [19]. Thus, it seems that directing attention (a top-down influence rather than an automatic reaction) can increase the neural activity in somatosensory-discriminative component of pain processing. Notably, a recent fMRI study also demonstrated that the perception of pain of others can be modulated by attentional and task demands [20].

**Figure 1:**
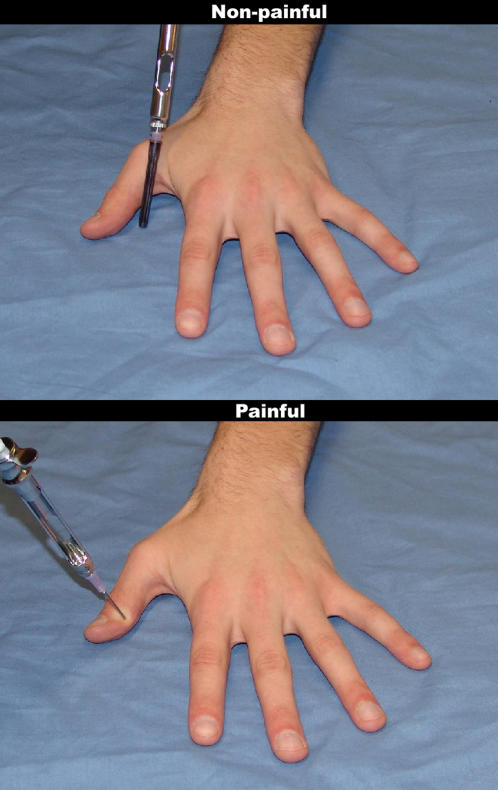
Examples for the stimuli used in the behavioral experiment and in fMRI experiment I. The upper image shows a needle covered by a black protector cap placed next to the hand (non-painful control stimulus). The lower image shows the (painful) injection of the same needle into the hand.

In the first fMRI experiment of this study, we therefore asked participants to either evaluate the sensory or the affective consequences of non-painful and painful situations (needle injections into different parts of a human hand, Figure 1). We expected that focusing on the sensory consequences of the inflicted pain would recruit somatosensory areas in a more pronounced way, whereas attending to affective aspects should result in stronger activation in areas coding the affective-cognitive dimension of pain (such as the anterior insula and the anterior medial cingulate cortex (aMCC)). Conceptually, this approach also poses the interesting question whether there are different ‘routes’ (i.e., neural pathways) when perceiving another person in pain, whether these pathways can be selectively activated, and to what extent they are similar to those involved in the first-hand perception of pain.

In the second fMRI experiment we explored the fact that emotions are malleable to various forms of cognitive regulation - such as suppression or (re)appraisal of the initial affective response [21]. Research in developmental psychology shows that one's ability to engage in emotion regulation positively relates to feelings of concern for the other person [22], [23]. Neuroscientific evidence concerning the modulation of the empathic response by cognitive appraisal and emotion regulation is, however, rather sparse. One study investigated the hemodynamic correlates of empathic feelings triggered by interacting with unfair targets [9]. The results showed signal reductions in areas coding the affective components of the empathic response and signal increases in reward/punishment-related brain areas. Another study recently demonstrated that the appraisal of others' pain is mediated by brain structures involved in stimulus evaluation and emotion regulation (such as the medial orbitofrontal cortex OFC and the right lateral prefrontal cortex [24]). Interestingly, this study neither revealed significant signal changes in sensory areas nor in areas thought to be part of the network supporting affective sharing (anterior insula and aMCC; however, activation in a more rostral part of the cingulate cortex was modulated by appraisal). Therefore it challenges the hypothesis that activation in this network indicates some sort of simulation of the other's actual emotional experience. It also shows that the top-down control exerted by appraisal does not seem to act upon early perceptual computations.

**Figure 2:**
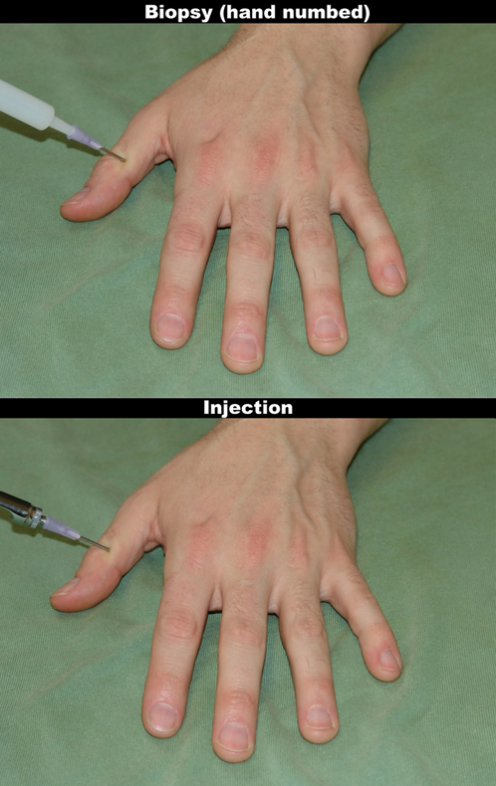
Samples for the stimuli used in fMRI experiment II. The upper image shows a (non-painful, but unpleasant) tissue biopsy from the numbed hand. The lower image show the (painful) injection of novocaine into the hand. Note the different types of syringes used in the two conditions, indicating their different functions.

The current experiment exposed participants to situations that normally would cause pain in both self and other (needle injections into a human hand). In some cases, however, the observer knew that the target's hand had been anesthetized in order to render the injection non-painful for the target (Figure 2). We expected the associated down-regulation of empathy to be accompanied by signal modulations in OFC and medial and lateral prefrontal areas, as well as in brain regions involved in self/other distinction. In addition, we anticipated significantly reduced activation in the affective components of the pain matrix, reflecting the absence of pain in the target.

## Results

### Behavioral experiment

Photographs depicting needle injections led to higher pain intensity and pain unpleasantness ratings than the photographs in which the needle was covered by the black protector cap (main effect *stimulus* (*painful* vs. *non-painful*), F(1,22) = 510.641, *P*<0.001, η^2^ = 0.959). In addition, the mean intensity and unpleasantness ratings were significantly different (main effect for *rating*, F(1,22) = 13.389, *P* = 0.001, η^2^ = 0.378), while no significant interaction term was found (*P* = 0.413). The following ratings (mean±S.D.) were obtained: intensity/painful 64.84±19.065; intensity/non-painful: 1.444±2.221; unpleasantness/painful: 69.033±14.225; unpleasantness/non-painful: 9.164±14.304). The Pearson correlation between intensity and unpleasantness ratings was *r* = 0.769 (*P*<0.001), showing that the two types of rating share about 50% of their variance. When the non-painful stimuli were excluded from this calculation, the correlation remained basically unchanged (r = 0.797) - indicating that the two stimulus dimensions have similar correlation for both painful and non-painful stimuli. On average, ratings were given within about 2.5 s (average response times for intensity and unpleasantness ratings 2.693 s and 2.767 s, respectively; no significant main effects or interaction for response times, *Ps*>0.153). The mean scores of the eight blocks revealed that ratings did not systematically decrease over the course of the experiment (non-significant main effect of the factor block: *P* = 0.410, η^2^ = 0.04; non-significant interaction block×rating, *P* = 0.335, η^2^ = 0.049).

### Functional MRI experiments

#### Dispositional measures

Results for the three questionnaires (Interpersonal Reactivity Index IRI [25], Emotional Contagion Scale ECS [26], Sensitivity to Pain Questionnaire SPQ [27]) and their subscales are documented in Table S1. Data for the IRI are well within published norms (as reported in detail in [5]), while the sample mean for the ECS was slightly below the norm average. SPQ sample means are comparable to a study collecting data from 96 normal controls [28]. Correlation coefficients (Pearson) reveal that the ECS correlates significantly with the IRI Fantasy scale (*r* = 0.513, *P* = 0.029), the IRI Empathic Concern scale (*r* = 0.469, *P* = 0.049), and the IRI Personal Distress scale (*r* = 0.545, *P* = 0.019). The discrimination score (P(A)) of the SPQ was inversely related to the Personal Distress scale (*r* = −0.504, *P* = 0.033), and positively correlated with IRI Perspective Taking (*r* = 0.519, *P* = 0.027). In addition, P(A) showed a significant correlation with the response bias value *B* of the SPQ (*r* = 0.648, *P* = 0.004). *B* also significantly correlated with IRI's Personal Distress subscale (*r* = −0.605, *P* = 0.008), and a trend towards significance was observed for ECS (*r* = −0.454, *P* = 0.059).

#### Pain ratings in the scanner

Similar to the behavioral experiment, photographs depicting injections led to significantly higher rating scores than images of the needle with the protector cap (main effect *stimulus*, F(1,17) = 348.815, *P*<0.001, η^2^ = 0.954). This was the case for both intensity and unpleasantness ratings (mean±S.D. for intensity/painful stimulus: 69.789±14.654; intensity/non-painful stimulus: 3.548±9.68; unpleasantness/painful: 71.237±13.65; unpleasantness/non-painful: 2.054±4.005). Neither the interaction term (*P* = 0.287) nor the main effect of rating were significant (*P* = 0.982). No significant change in scores across the two imaging runs was observed, indicating the absence of strong habituation.

**Figure 3:**
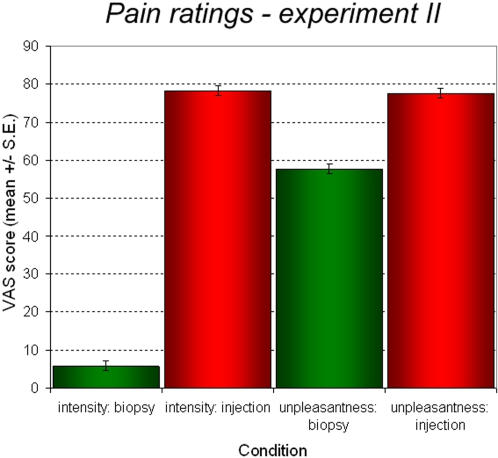
Behavioral data from fMRI experiment II. Injections led to high intensity and unpleasantness ratings, while rated pain intensity for the numbed hand stimuli is close to zero. Note also that although the unpleasantness ratings for the numbed hand stimuli are significantly smaller than for the injection stimuli, they are substantially high and significantly different from zero.

In the second fMRI experiment (Figure 3), injections into a numbed hand were perceived as non-painful, but considerably unpleasant – while injections into a hand that was not numbed were perceived as both highly painful and unpleasant (main effect numbed vs. non-numbed: F(1,16) = 404.426, *P*<0.001, η^2^ = 0.962; rating/intensity vs. unpleasantness: F(1,16) = 90.444, *P*<0.001, η^2^ = 0.850; significant interaction appraisal×rating: F(1,16) = 145.33, *P*<0.001, η^2^ = 0.901; significant post-hoc test contrasting injections into non-numbed vs. numbed hands for unpleasantness ratings, F(1,16) = 90.444, *P*<0.001). Again, scores did not significantly change over the course of the experiment. Note that due to excessive movement during experiment II, one participant had to be excluded from all analyses.

### fMRI experiment I – effects of evaluative focus

#### Perception of Pain vs. NoPain

**Figure 4:**
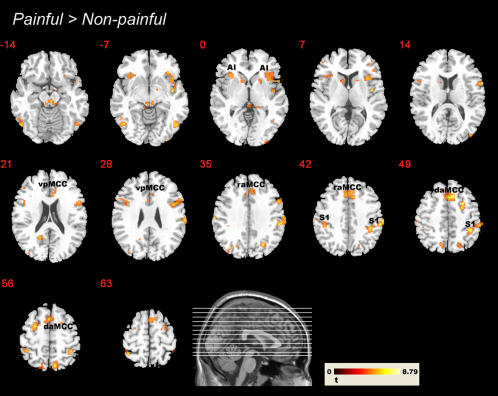
Significant clusters from the random effects contrast painful>non-painful (intensity and unpleasantness rating trials pooled) of fMRI experiment I, displayed on a high-resolution structural MRI template in MNI space (used in all figures, displayed in neurological convention; red numbers indicate slice number). The anatomical labels designate the approximate location (in the rfx average) of the functional ROIs (see text for abbreviations). Threshold *P* = 0.01 (FDR-corrected), *k* = 10.

In order to assess the neuro-hemodynamic response to the perception of painful situations, we contrasted activation during painful injections with those where the needle was covered by the black cap (pooled for the two rating conditions, i.e., All_painful>All_Non-painful). This contrast indicated the involvement of large portions of the pain matrix [29], [30]. Activation clusters were detected in areas coding the affective, the sensory and the motor aspects associated with nociception (Figure 4). Brain areas involved in affective-motivational coding included the dorsal and ventral aMCC, bilateral anterior insula, and right middle insula. Large activation clusters extending from supramarginal gyrus into the postcentral gyrus reflect the involvement of primary and higher-order somatosensory areas (Areas 1 and 2, Area OP4, bilaterally; all areas defined based on cytoarchitectonic probability maps from the Anatomy Toolbox; [31]). Bilateral motor activations were observed in cortical, basal ganglia (striatum) and cerebellar motor areas (rostral supplementary motor area and cingulate motor area, dorsal lateral premotor areas, caudate nucleus and putamen). In addition, strong bilateral involvement of the supramarginal gyri and of inferior frontal gyri (ventral premotor cortex, pars opercularis, Area 44) indicated the contribution of areas associated with the anticipation of action consequences. Activations were also found in the thalamus, in right medial frontal gyrus, and in the superior part of the periaqueductal grey. The consistency of the group analysis was confirmed by analyses on the single-subject level – as the five functional regions of interest (ROIs) described in the Material & Methods section were clearly activated in the majority of participants. Table S2 shows the peak coordinates of these ROIs for each individual participant.

#### Intensity vs. Unpleasantness of pain

To investigate whether evaluating the sensory or the affective consequences of painful stimulation leads to differential activation in the pain matrix we assessed the interaction contrasts of our design. The contrast *Intensity (Painful>Non-Painful)>Unpleasantness (Painful>Non-Painful)* yielded several significant clusters in the sensori-motor network identified by the comparison of painful and non-painful stimuli. The strongest activation modulation was obtained in right postcentral gyrus, contralateral to the stimulated target's hand. This indicates an important role of somatosensory processing in differentiating between sensory and affective stimulation consequences. The involvement of areas associated with anticipating action consequences was indexed by activation clusters in inferior parietal cortex/supramarginal gyrus and ventral premotor areas (see Table 1; Figure S1). Notably, the two types of rating did not modulate activation in the anterior insular cortices. However, activation differed in mid- and posterior insular cortices - i.e., in areas that are specifically involved in the first-hand experience of pain. The increased thalamic activation might be related to a similar mechanism (see discussion). In addition, stronger activation was observed in the aMCC at the transition zone from the cingulate gyrus to the superior frontal gyrus. The reverse interaction [*Unpleasantness (Painful>Non-Painful)>Intensity (Painful>Non-Painful)*] only yielded a significant cluster in visual cortex (right lingual gyrus, MNI 23/−82/8). Lowering the threshold to *P* = 0.005, *k* = 5, revealed additional clusters in the right cerebellum, and in subcallosal cingulate cortex (see Table 1).

**Table 1:** Significant differences resulting from the interaction contrasts Intensity (Painful>Non-painful)>Unpleasantness (Painful>Non-painful) and vice versa.

	L/R/M	k	t	x	y	z
**Interaction: Intensity>Unpleasantness**						
Precuneus	L	43	6.50	−22	−50	12
Precuneus	R	235	5.72	14	−44	38
×Precuneus	L		4.24	−10	−52	38
×Precuneus	M		3.80	4	−50	40
Angular Gyrus	R	174	6.00	50	−64	48
Angular Gyrus	L	21	3.73	−48	−66	40
Inferior Temporal Gyrus	L	8	4.02	−52	−4	−38
Inferior Temporal Gyrus	R	21	3.89	58	−22	−28
Middle Temporal Gyrus	R	186	4.82	56	−70	20
Angular Gyrus	R		4.80	46	−64	24
×Middle Temporal Gyrus	R		4.37	38	−54	18
Inferior Temporal Gyrus/Temporal Pole	R	39	5.02	48	0	−42
×Inferior Temporal Gyrus/Temporal Pole	R		4.33	52	−6	−38
Superior Temporal Pole/fronto-insular cortex	68	68	4.61	30	8	−24
Middle/Superior Temporal Gyrus	L	9	3.88	−66	−44	8
Calcarine sulcus	M	372	5.61	4	−52	14
Lingual gyrus	R		4.46	12	−50	4
Ventral Precuneus	M		4.23	−2	−66	30
Fusiform Gyrus	L	5	3.64*	−32	−66	−10
Supplementary Motor Area	M	8	3.79	6	−22	48
×Supplementary Motor Area	M		3.42*	10	20	68
Precentral Gyrus	R	10	3.65*	24	−24	76
Rolandic Operculum	L	7	3.15*	−38	−14	24
Superior Frontal Gyrus	M	32	3.74	4	26	64
Superior Frontal Gyrus	R	5	3.60*	20	64	8
Middle Frontal Gyrus	R	19	3.57*	40	20	54
Inferior Frontal Gyrus	L	5	3.23*	−52	34	14
Inferior Frontal/Orbitofrontal Cortex	R	11	3.42*	48	32	−8
Inferior Frontal/Orbitofrontal Cortex	R	8	4.20	32	36	−8
Inferior Frontal Gyrus/Orbitofrontal Cortex	L	13	4.05	−44	30	−18
Cerebellum/Lingual Gyrus	R	26	4.03	12	−44	−10
Cerebellum	R	11	4.06	22	−24	−28
Parahippocampal area/Amygdala	R	17	3.78	24	0	−26
**Interaction: Unpleasantness>Intensity**						
Insula	L	7	4.77	−30	24	8
Anterior Insula	R	8	3.40*	30	30	6
Rolandic Operculum/posterior Insula	R	7	3.72	44	−6	10
Cerebellum	R	8	4.58	12	−62	−44
Cerebellum	M	10	3.80	8	−80	−44
Cerebellum	R	25	4.62	28	−72	−50
Caudate/Putamen	M	10	3.94	−8	4	−10
Orbitofrontal Cortex	R	10	4.11	22	44	−10
Superior Frontal Gyrus/Precentral Gyrus	L	18	4.09	−22	−12	54
Middle Occipital Gyrus	L	27	4.01	−40	−90	−4
Supramarginal Gyrus	R	17	3.36*	44	−32	38
Inferior Parietal Cortex	L	7	3.28*	−24	−56	40
Inferior Parietal Cortex	R	9	3.27*	30	−46	44

Notes: Voxel threshold *P* = 0.001 (uncorrected), cluster size threshold *k* = 5. * *P* = 0.005, *k* = 5; stereotactic coordinates and t-values are provided for the local voxel maximum of the respective cluster. x = sub-peaks of a cluster, L = left hemisphere, R = right hemisphere, M = medial activation, k = number of activated voxels in cluster; areas (in brackets, e.g. OP4) determined based upon cytoarchitectonic maps provided in the Anatomy Toolbox.

#### Relationship between dispositional and behavioral measures and brain activation

Emotional contagion scores correlated significantly with activation (All_painful>Baseline) in the affective-motivational component of the pain matrix, including bilateral anterior insula and two distinct clusters in aMCC. While insular activation overlapped almost perfectly with the clusters detected by the contrast of painful with non-painful stimuli, activation in aMCC was considerably more rostral. Additional significant correlations were observed in bilateral supramarginal gyri, the precuneus, and various visual areas.

The correlation between the IRI empathic concern subscale and activation differences between painful and non-painful trials (All_painful>All_non-painful) yielded significant positive correlations in bilateral dorsal premotor cortex, left ventral premotor cortex, left somatosensory cortex, in medial bilateral posterior precuneus and in bilateral fusiform gyrus. No significant clusters were detected in insular or cingulate cortices, even when lowering the threshold to *P* = 0.005. However, an additional large cluster in the right supra-marginal gyrus was detected at the lower threshold (stereotactic coordinates x/y/z = 56/−37/41).

Correlation analyses with pain ratings indicated an important role for posterior inferior temporal gyrus and bilateral ventral premotor cortex (Area 45, pars triangularis) in evaluating the amount of pain and its unpleasantness. Pain intensity ratings were additionally associated with activation in contralateral precentral gyrus, and in dorsal posterior cingulate gyrus in a region involved in visuo-spatial attention [32]. Significant correlations in supramarginal gyrus extending into SII suggest that focusing on the affective consequences selectively recruited this region (see Table S3 for a complete list of correlations).

### fMRI experiment II – effects of cognitive appraisal

#### Whole brain analyses

The aim of fMRI experiment II was to assess how activity in the pain matrix is modulated by the appraisal of a seemingly painful and aversive, but actually non-painful situation. According to the information given to the participants, the novocaine injections and the subsequent biopsies on the numbed hand differed in one crucial aspect: While the numbing of the target's hand resulted in a complete loss of pain somatosensation, the targets still experienced unpleasantness and discomfort due to the surgical procedure. As indicated above, the behavioral data show a clear effect of this instruction on the pain ratings since putative anesthesia reduced imputed pain. At the neural level, we hypothesized a similar differentiation in neural activity between intensity and unpleasantness ratings. Brain activation in areas of the pain matrix was expected to be different during intensity ratings while unpleasantness ratings should hardly result in activation differences - since both the injections into the numbed and into the non-numbed hand were supposed to be unpleasant for the target. Statistically, this hypothesis was assessed by the interaction terms between the factors rating and stimulus.

**Figure 5:**
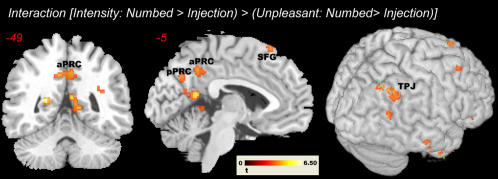
Significant clusters in anterior and posterior precuneus (aPRC and pPRC) and in the right temporo-parietal junction (TPJ) revealed by the interaction contrast (Intensity: Numbed>Injection)>(Unpleasant: Numbed>Injection). Threshold *P* = 0.001 (uncorrected), *k* = 5.

The interaction contrast [Intensity: Numbed hand>Painful Injection)>(Unpleasantness: Numbed Hand>Painful Injection] yielded significant clusters in the precuneus and bilaterally in the temporo-parietal junction (see Figure 5). Interestingly, these effects resulted from a relative difference in deactivation between conditions – with the target contrast (numbed hand>baseline during intensity trials) being the only condition that showed activation and all the other conditions showing deactivation. Activation differences were also detected in middle and anterior inferior temporal gyrus, in particular in both temporal poles – a region supposedly involved in linking perceptual information with emotional and visceral responses as well as in mentalizing [33]. In the frontal lobe, activation differed in medial and in superior frontal gyrus as well as in lateral OFC. There were no significant clusters in occipital primary or secondary visual areas, not even when lowering the threshold to *P* = 0.05 (uncorrected). The reverse interaction ((Unpleasantness: Numbed hand>Injection)>(Intensity: Numbed hand>Injection)) revealed significant signal modulation in the left anterior insula, the cerebellum, OFC cortex and the basal ganglia. Lowering the threshold to *P* = 0.005 yielded additional clusters in right anterior insular cortex, and in the inferior parietal cortex/supramarginal gyrus. See Table 2 for a complete list of significant activations.

**Table 2:** Significant differences resulting from the interaction contrasts Intensity (Numbed>Non-numbed)>Unpleasantness (Numbed>Non-numbed) and vice versa.

	L/R/M	k	x	y	z	t-value
**Interaction: Intensity>Unpleasantness**						
Postcentral gyrus (Area 2)	R	24	26	−44	48	6.38
Postcentral gyrus (Area 3a)	L	36	−18	−36	50	5.55
Postcentral Gyrus (Area OP4)	L	45	−62	−14	18	4.56
×Postcentral Gyrus (Area OP4)	L		−60	−22	28	4.37
Precentral Gyrus (Area 6)	R	10	26	−18	62	4.97
Superior Temporal Lobe	L	12	−42	−8	−12	4.17
Superior Temporal Pole	L	16	−40	4	−20	4.45
Inferior Temporal Gyrus	L	127	−60	−62	−6	6.34
×Inferior Temporal Gyrus	L		−50	−58	0	4.86
×Inferior Temporal Gyrus	L		−52	−62	−10	4.12
Supramarginal Gyrus	R	67	58	−34	32	5.13
×Supramarginal Gyrus	R		58	−28	26	4.10
×Supramarginal Gyrus	R		54	−36	24	3.95
Precuneus	L	51	−12	−60	56	5.65
Precuneus (extending into Area 4a)	M	14	4	−40	52	5.36
Inferior Occipital Gyrus	R	26	44	−76	−6	5.10
Fusiform Gyrus	L	22	−30	−44	−18	4.81
Lingual Gyrus/Calcarine Sulcus (Area 17)	R	22	14	−56	8	4.72
Calcarine Sulcus	L	11	−18	−56	10	4.36
Lingual Gyrus (Area 17)	R	21	24	−50	−4	4.43
Thalamus	R	10	16	−6	6	4.39
Hippocampus	R	20	28	−18	−10	5.88
Hippocampus	R	10	40	−2	−20	4.09
Hippocampus	R	11	36	−16	−12	3.97
Parahippocampal Gyrus	M	22	−6	−18	−30	4.92
×Pons	M		4	−20	−26	4.76
Cerebellum	R	39	16	−64	−22	4.95
×Cerebellum	R		24	−74	−20	4.41
Cerebellum (Vermis)	L	10	−12	−56	−50	5.12
Cerebellum (Crus)	L	29	−34	−52	−34	4.42
Cerebellum	R	18	42	−46	−44	4.38
Superior Frontal Gyrus	R	14	26	2	64	4.81
Inferior Fontral Gyrus/Operculum (Area 44)	R	67	48	10	2	4.59
×Inferior Fontral Gyrus/Operculum (Area 44)	R		52	8	10	4.57
×Midinsular Cortex	R		52	10	−6	4.23
Rolandic Operculum (Area 44)	L	10	−54	10	0	4.23
Medial Insular Cortex	L	15	−40	0	2	4.14
Anterior Medial Cingulate Cortex	M	68	0	24	36	5.07
Anterior Medial Cingulate Cortex	R	10	12	26	32	4.53
Anterior Medial Cingulate Cortex	M	28	−4	10	42	4.47
**Interaction: Unpleasantness>Intensity**						
Calcarine Sulcus	R	45	22	−82	6	5.68
Cerebellum	R	19	18	−86	−36	4.85
Subcallosal Cingulate Cortex	M	18	−6	20	−4	4.31

Notes: see Table 1 for specifications and abbreviations.

**Figure 6:**
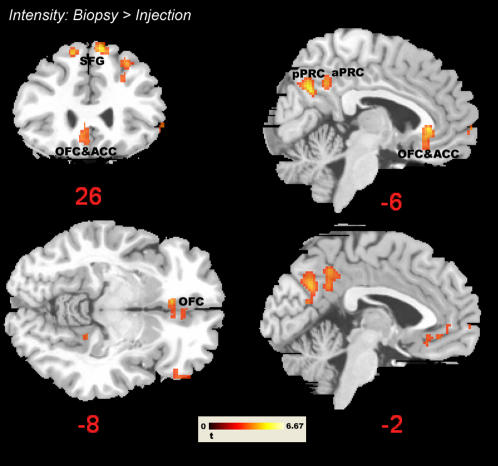
Additional clusters in orbitofrontal cortex (OFC) and subcallosal/perigenual ACC when contrasting the biopsy with the injection condition during pain intensity ratings (numbed>injection; intensity rating trials only). Threshold *P* = 0.005 (uncorrected), *k* = 5.

In addition, we scrutinized the contrasts Numbed Hand>Painful Injection and Painful Injection>Numbed Hand for those trials in which participants evaluated pain intensity. This analysis was performed to capture differences that might have been missed by the interaction analyses – whose results also depend upon the assumption of no or negligible differences for the unpleasantness evaluations of injections and numbed hands. This analysis basically confirmed the results of the interaction contrasts - showing that the latter mainly resulted from of a lack of differences for unpleasantness ratings along with different hemodynamic responses during the intensity ratings. However, a few additional clusters were detected (see Figure 6). The contrast Intensity: Numbed>Injection revealed significant clusters in perigenual anterior cingulate cortex (ACC), subcallosal ACC, medial OFC, bilateral superior frontal gyrus, and in the pars orbitalis and triangularis of the right inferior frontal gyrus. Lowering the threshold to *P* = 0.005 (uncorrected) yielded additional clusters in medial OFC and a small cluster encompassing right pre- and postcentral gyrus (Areas 3 and 4; see Figure 6 and Figure S2). The reverse contrast (Intensity: Injection>Numbed; Figure S3) indicated additional activation differences in bilateral dorsal and ventral premotor cortex, in bilateral superior parietal lobe and bilateral lateral precuneus, and in several thalamic nuclei.

Furthermore, in order to assess the reproducibility of results across the two fMRI experiments, we compared the results of the contrasts Painful Injection>Baseline (experiment II) and Painful stimuli>Baseline (experiment I; both contrasts pooled for intensity and unpleasantness ratings). This comparison indicated excellent reproducibility of results, with experiment II yielding basically the same findings as experiment I for the painful injections.

#### ROI analyses – effects of cognitive appraisal

We specifically assessed activation in six ROIs (three in medial cingulate cortex, bilateral anterior insulae, contralateral primary somatosensory cortex) hypothesized to reflect different kinds of affective information processing during empathy for pain. These analyses tested hypotheses about activation differences in *a priori* and functionally defined areas with higher sensitivity. In addition, they were used to investigate the time-courses of signal changes without assumptions about the shape of the hemodynamic response. Activation of the anterior insula during affective processing in general as well as during the perception of pain in others is well-documented and seems to be related to interoceptive awareness and affective evaluation [34]. The same applies for MCC activation, with different subregions being related to distinct processes. While activation in ventral posterior MCC (vpMCC) is usually associated with interoceptive awareness and monitoring of bodily responses [35], neurons in dorsal anterior MCC (daMCC) seem to be involved in motor processing triggered by the observation of pain [36]. Finally, activation in rostral anterior MCC (raMCC) seems to reflect evaluation processes related to the aversive consequences of noxious stimulation.

**Figure 7:**
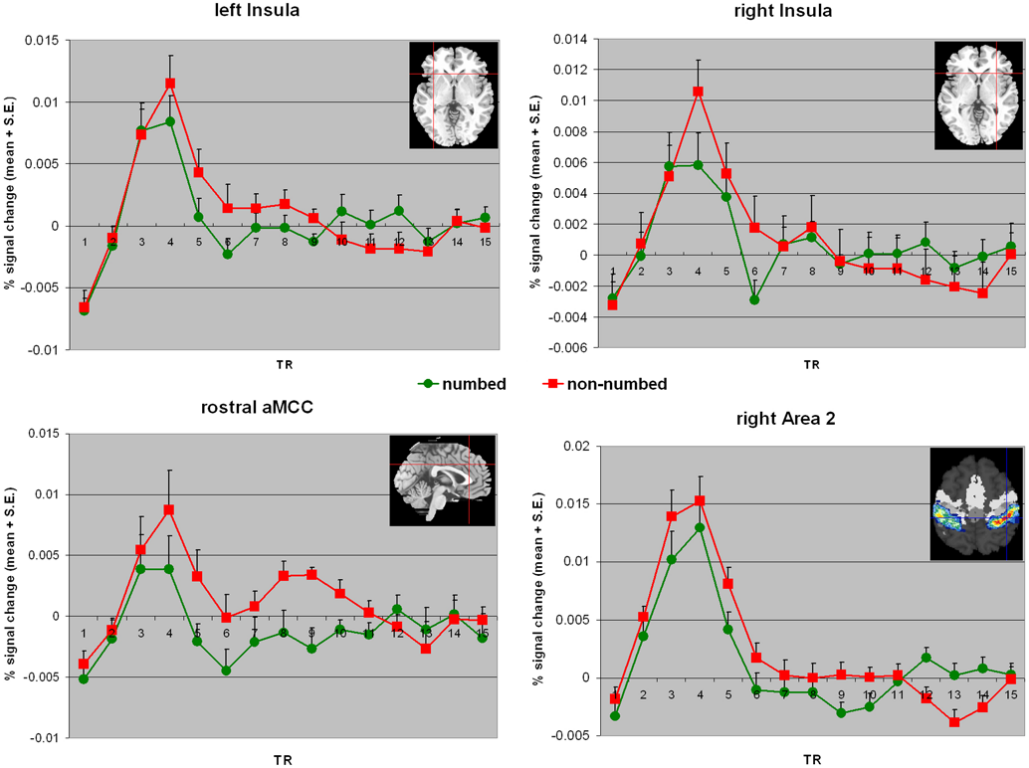
Time-courses in the ROIs (anterior insulae, rostral aMCC and contralateral somatosensory cortex/Area 2) analyzed in fMRI experiment II. Note that all areas show a significant hemodynamic response during both the injection and the numbed hand stimuli. Significant differences as determined by linear contrasts are indicated by asterisks (** = P<0.05, * P<0.10, see text for details).

All ROIs indicated a ‘typical’ hemodynamic response peaking around five to seven seconds and returning to baseline levels around fifteen to twenty seconds post stimulus. Signal changes were similar for both the biopsies and the injection stimuli. Significant interaction effects (stimulus×rating), however, were observed in raMCC where higher signals for injection stimuli rated for pain intensity were accompanied by non-differing responses for unpleasantness ratings (F(1,13) = 5.069, *P* = 0.042). In addition, there was a trend towards a significant interaction for the right anterior insula (F(1,16) = 3.45, *P* = 0.082). All other linear contrasts were non-significant (all *Ps*>0.152). When contrasting only trials rated for pain intensity, the effect for the right insular ROI was significant (F(1,16) = 6.34, *P* = 0.023) – being related to reduced activation during biopsies on the numbed hand evaluated for pain intensity (Figure 7). In addition, there was a trend towards significance in contralateral somatosensory cortex (F(1,16) = 3.755, *P* = 0.07), reflecting higher activation during painful injections. The time-course analyses also revealed an interesting signal time-course for the rostral aMCC cluster - which showed a bimodal signal change with a second hemodynamic response about 9 image volumes (TRs) after stimulus onset for the painful injections (in both rating conditions, see Figure 7). A post-hoc comparison of TRs 9 to 11 contrasting non-numbed and numbed trials (pooled for the two rating conditions) revealed a significant difference for this ‘late response’ (F(1,13) = 6.96, *P* = 0.02).

#### Relationship between dispositional and behavioral measures and brain activation

##### Pain ratings

We hypothesized that the degree to which a participant showed a better behavioral differentiation between the numbed and non-numbed stimulus conditions when evaluating pain intensity would correlate with stronger signal differences in the pain matrix as well as in regions involved in emotion regulation and evaluation of stimulus valence. We therefore correlated the signal difference between numbed hand and injection trials (numbed>non-numbed, intensity trials only) with the difference in intensity ratings for numbed and non-numbed stimuli. This revealed a number of significant correlations in a network that largely overlapped with the one identified by the interaction contrast and additionally included a number of areas of the pain matrix (see Table S4).

##### Perspective taking

A similar result was expected when correlating the scores of the IRI perspective taking subscale with the activation differences between numbed and non-numbed stimuli (again, for intensity trials only). This expectation was largely confirmed, as the analysis revealed a very similar network as the correlation analysis computed with the pain rating differences. Results differed, however, with respect to areas involved in self-awareness and mentalizing such as the posterior precuneus, temporo-parietal junction (TPJ) or medial prefrontal/paracingulate cortex, which – contrary to our expectations - did not correlate with the perspective taking scores (Table S4).

##### Emotion Contagion

Here we assessed whether emotion contagion scores were inversely related to the activation difference between intensity-rated numbed and non-numbed trials. Our hypothesis was that a higher susceptibility to emotion contagion (and thus a stronger automatic or bottom-up driven reaction to even the non-painful stimuli) would result in lower activation differences in sensorimotor areas and in areas of the pain matrix. This hypothesis was partially confirmed by significant correlations in medial primary/premotor cortex (Areas 4 and 6) and in inferior parietal areas (supramarginal and angular gyri). However, no correlations were observed for insular or cingulate activations.

## Discussion

The aim of this study was to investigate how top-down control mechanisms modulate the neural underpinnings of empathy for pain. We assessed (1) whether focusing on the sensory or the affective consequences of another's pain distinctly recruits neural pathways involved in sensory-discriminative and affective-motivational processing; and (2) which brain structures subserve the appraisal and down-regulation of empathic responding when witnessing injections into the numbed hand of another person. In addition, we explored the influence of individual differences in empathic concern, emotion contagion and the sensitivity to pain on this modulation. We will first discuss the individual results of each experiment, and then conclude with a general discussion.

### Behavioral experiment and pain ratings

Results from the behavioral experiment indicate that participants were able to correctly evaluate the sensory and affective consequences of painful needle injections. Further, the absence of systematic changes in ratings across the course of both fMRI experiments demonstrates that behavioral evaluations were not affected by habituation effects. Interestingly, the correlation between intensity and unpleasantness ratings was similar to correlations obtained during the *first-hand* experience of pain e.g., [37], [38]. This suggests that ratings of one's own and another's pain might share some common evaluative processes - at least in terms of their behavioral outcomes.

### The role of sensory and affective components in empathy for pain

A growing number of neuroimaging studies reliably documents that witnessing pain in others activates a similar network as the first-hand experience of pain [12], [34 for reviews]. Consistent activation in bilateral anterior insula and in dorsal and ventral aspects of aMCC documents the importance of brain areas involved in the affective-motivational coding of pain. In addition our results generate two crucial insights. First, we observed consistent activation in bilateral somatosensory areas, with activation being more pronounced in the right hemisphere – i.e., contralateral to the stimulated hand. Second, our results demonstrate an important role of ventral premotor and rostral inferior parietal cortex (supramarginal gyrus, inferior parietal lobule, encompassing the intraparietal sulcus; Area hlP2) in the perception of pain in others. These activations can be interpreted within a conceptual framework stressing the importance of serial predictions and event sequencing to anticipate and understand the actions of others (e.g., [39]). Understanding the consequences of the shown actions is clearly required in both fMRI experiments as participants were asked to infer the consequence of the needle injections and to evaluate them in a fine-grained way using a visual analogue scale (VAS). Following the logic of this framework, activation in inferior parietal areas may result from the object-related actions displayed (with the object being the pricked hand in the current case), while ventral premotor activation is related to anticipating the resulting sensory and affective consequences of the displayed action. This is in line with increased functional connectivity of ventral premotor clusters with medial cingulate areas during the rating of pain in others observed in another study [20]. Note also that activation in ventral premotor cortex positively correlated with the pain intensity ratings (Table S3). In addition, part of the clusters in inferior parietal cortex might be related to the coding of nocifensive movements, and the visuospatial encoding of noxious threats [40], [41].

The consistent activation of primary somatosensory cortex can be seen in two, not mutually exclusive ways. First, it might reflect the unspecific co-activation of somatosensory representations by neurons in inferior parietal and premotor cortex that are involved in understanding the action's consequences and by means of a feedback loop activate their associated somatosensory representations. Alternatively, somatosensory representations might be involved more specifically by locating the ‘impact’ point of the aversive object, hence playing a more causal role in coding the action's sensory and aversive consequences. Depending upon where the hand or finger is punctured, this will inform the observer about the resulting pain intensity or unpleasantness. Partial support for this hypothesis comes from studies on the anticipation of touch (e.g., [42], [43]) as well as from the common coding theory which posits that actions are coded in terms of their perceivable effects [44]. Which one of these hypotheses is correct and therefore which functional role somatosensory representations play in understanding another's emotion should be determined by future studies. Interestingly, a recent event-related potentials (ERPs) study also reports modulation of somatosensory-evoked potentials with pain intensity but not with pain unpleasantness [17], supporting our finding that focusing on the consequences of painful stimulation reliably triggers activation in a neural network involved in action understanding and somatosensation. Note also that both the somatosensory ERPs and our hemodynamic responses cannot be explained by the observation of touch alone as stimuli displaying non-painful touch were used as control stimuli in both experimental paradigms.

### Correlations between brain activation and dispositional measures

The correlation analyses yield interesting clues as to what aspect of empathic responding our experimental design triggers, and to which psychological processes activations in the pain matrix might be related to. Current neurobehavioral models of empathy (e.g., [12], [13], [45]) emphasize the contribution of both automatic and controlled processes to the conscious experience of empathy. The emotion contagion questionnaire assesses an individual's susceptibility to automatically mimic another's behavior – a mechanism that is also found in phylogenetically older species (e.g., [46], [47]). Conversely, the empathic concern scale measures the more sophisticated aspect of empathy under cognitive control. Hence, the significant correlations in regions involved in affective-motivational as well as in motor processing with emotional contagion suggest that activation in these areas might be related to more bottom-up driven processes, such as motor resonance and affective sharing. To the contrary, the empathic concern scale does not covary with activations in the anterior insula and ACC. Instead, the pattern of significant correlations in prefrontal cortex and OFC probably relates to the more cognitive components of empathy assessed by this scale. Note though that studies which created a more direct social interaction between observer and target (e.g., [8], [24]) also found correlations in affect-related areas.

### Effects of focusing on sensory vs. affective consequences of pain

There is an ongoing debate about whether perceiving and understanding the pain of others is mediated by somatosensory or by affective representations. While two TMS studies [15], [16] and a recent ERP study [17] suggested involvement of sensorimotor processing, most fMRI results support the idea that the empathizers' response relies upon representing the affective rather than the sensory consequences of the other's pain. One explanation for these discrepancies might be the focus of attention in the fMRI *vs.* the other studies. The instruction of the TMS studies made participants explicitly reason about the sensory consequences of the stimulation and directed their attention to the specific body part that was getting punctured. In addition, as the stimuli were short video-clips, participants could predict the location and the time of impact of the needle on the body surface. This reasoning about the spatio-temporal and the sensory consequences of the stimulation might have triggered increased activation in the sensory-motor system. In our experiments, therefore, we asked participants to focus on either the sensory or the affective consequences of painful stimulations.

The different instructions recruited distinct neural networks. Focusing on pain intensity was associated with increased signal in contralateral somatosensory cortex (S1) and in contralateral premotor cortex. This indicates a stronger contribution of sensorimotor representations to assessing the sensory consequences of pain. A more immediate representation of the target's actual sensory-somaesthetic experiences is also suggested by stronger activations in areas involved in coding the immediate and first-hand sensory consequences of pain - such as the posterior parts of the insula, the thalamus or the hippocampus. The contralateral middle insular cortex has intrinsic connections to the basal ganglia, and a meta-analysis of neuroimaging studies shows that it is most consistently activated during the first-hand experience of pain [48] – suggesting a specific role in coding the sensory-motor aspects associated with pain. This part of the insula also shows stronger signal changes when participants imagine pain from a first-person perspective [3], [24], [49]. In addition, electrical stimulation of the posterior part of the insula evokes painful sensations while stimulation of more anterior parts does not [50]. Activations in the thalamus and the hippocampus supplement the view that evaluating for pain intensity leads to a more immediate and direct experience of the target's sensory and affective experience. Notably, the hippocampus might reflect memory-related processes activated during both the first-hand and the vicarious perception of pain [3], [51]. Focusing on the sensory consequences also resulted in stronger activations in the action anticipation network outlined above (inferior parietal cortex and ventral premotor cortex), as well as in two distinct clusters in the anterior cingulate. The more rostral one of these clusters is located in the transition zone between superior frontal and anterior cingulate gyrus. This region responds selectively to increases in stimulus intensity and in subjective pain intensity [52]. Conversely, the more caudal cluster can be assigned to the cingulate motor area and most likely supports motor preparation and motor mobilization not specific to pain but to stimulus intensity.

Focusing on the unpleasantness of pain did not lead to significant changes in any brain regions, except for small clusters in visual cortex and in subcallosal ACC. The only indicator of increased affective representations is the cluster in subcallosal ACC. Neurons in this area have been associated with processing of negative affect [53] and this area has many connections to subcortical autonomic centers. Hence, our initial prediction that the perception of pain in others specifically recruits the sensory and the affective parts of the pain pathways only holds for the sensory realm. Activation during intensity ratings suggests higher personal involvement during that condition. Therefore, even though participants were not explicitly instructed to focus on the affective consequences, this higher involvement may lead to an implicit activation of the affective-motivational parts of the pain matrix to an extent that was similar as during the explicit unpleasantness ratings. Alternatively and in line with the findings of experiment II, the presentation of the aversive stimuli along with the requirement to evaluate their painful consequences might by default activate the affective components of the pain matrix - irrespective of the cognitively mediated attentional focus. Note also that although activation in some somatosensory areas was higher during intensity ratings, unpleasantness ratings led to similar activations of somatosensory cortex – indicating that the classical separation of a ‘sensory’ and an ‘affective’ neural pathway may not apply to the evaluation of pain in others. Interestingly, the significant correlation of unpleasantness ratings with activation in secondary somatosensory cortex also suggests a role of somatosensory representations in rating affective stimulation consequences.

Taken together, the results of fMRI experiment I replicate and extend previous findings concerning empathy for pain by showing a stronger involvement of neural structures involved in action anticipation and somatosensation when focusing on the sensory consequences of mechanically induced pain [34], [54], [55]. The activation pattern suggests that attending to pain intensity leads to higher personal involvement as indicated by stronger activation of brain areas associated with action understanding, noxious threat evaluation and nocifensive reactions. This might result from pain intensity being the more crucial variable from a survival point of view - as it is more important to evaluate the actual injury inflicted than its affective correlates or ‘side effects’.

### The role of appraisal in empathy for pain

Within the framework of appraisal theory [56], it is the interpretation of an external or internal event that determines its affective consequences and the associated experiences. This theory emphasizes the importance of cognitive processes for emotional responses, posits their malleability and flexibility, and highlights the role of re-appraisal in coping with adverse life events. Accordingly, identical stimuli can result in surprisingly different affective reactions - depending upon stimulus context and the appraisal and coping mechanisms an individual has developed. This also applies for empathic reactions which are a compound of the eliciting stimulus and the interpretation of that stimulus by the empathizer. Recent findings from an fMRI study support such a view [9], showing activation modulation in areas involved in affective processing and valence evaluation (insula and orbitofrontal cortex) with different appraisal.

An important distinction in neural investigations of appraisal and emotion regulation is to determine areas that are the *sources* of regulation as well as their *sites*. *Sources* of regulation are supposed to implement the actual processes allowing for emotion regulation - for example, by means of executive control or by (re)evaluations of the stimulus or event valence. These processes affect (indirectly or directly) the *sites* representing the actual affective state. For example, it has been shown that anxiety reduction is mediated by rostro-lateral prefrontal areas as the sources and medial prefrontal/anterior cingulate areas as the sites of emotion regulation [57].

Based upon neuroimaging evidence and neuronal connectivity, we predicted the sources of modulation to be prefrontal areas involved in valence judgments and executive control (medial and lateral OFC, medial prefrontal cortex), dorsal and rostral areas of the MCC (evaluative and motivational processing), as well as areas relevant for self/other distinction and mentalizing – such as the medial precuneus, the temporo-parietal junction and the temporal poles. Reduced activity, on the other hand, was expected in the network coding affect such as bilateral anterior insula, bilateral amygdalae, as well as the ventro-medial portion of aMCC. In addition, we explored whether top-down control affects neural processing already at an early perceptual stage, which would result in reduced neural activity in areas involved in visual and somatosensory perception.

The behavioral data showed a clear interaction between the stimulus type and the type of rating that participants had to perform. While perceiving numbed vs. non-numbed hands resulted in clearly different pain intensity ratings, this effect was significantly reduced for unpleasantness ratings because – in line with the cover story - the biopsies on the numbed hand were evaluated as unpleasant for the target. This dissociation was associated with activation in areas involved in self/other distinction (precuneus and temporo-parietal junction), emotion regulation and valence evaluation (medial and superior frontal gyrus, OFC), and in action anticipation (right ventral premotor cortex). Such activation modulations can be attributed to the *sources* of appraisal processes. We suggest that the signal changes in the precuneus and the temporo-parietal junction reflect the requirement to distinguish one's own prepotent response to the sight of an aversive event from the knowledge about the actual effects for the shown target. Both the precuneus and the temporo-parietal junction have been associated with processes of self/other distinction, self-awareness and agency. The precuneus has widespread connections to a number of cortical and sub-cortical areas, including the posterior and anterior cingulate cortex and areas involved in motor control. This pattern of connectivity along with neuroimaging evidence on resting state metabolism and self-referential actions suggests a dominant role of this structure in self-awareness [58]–[60]. The precuneus also has reciprocal connections to a region initially labeled as parieto-temporo-preoccipital cortex [61] and coined as the TPJ in recent neuroimaging studies. The TPJ is a heteromodal association cortex associated with the processing of phenomenological and cognitive aspects of the self [62]. A recent meta-analysis documented that the TPJ is not only involved in various high-level cognitive phenomena such as empathy and theory of mind but also in lower-level computations [63]. The putative basis for these phenomena are neural computations related to updating and reorienting attention due to violations of expectations and the detection of change. Such a mechanism was also required in the current study where the displayed situation does not result in the aversive consequences it would bear under normal circumstances.

The involvement of medial and lateral areas of the dorsal medial prefrontal cortex, on the other hand, seems to be associated with cognitive and executive control processes. Areas in prefrontal cortex have been repeatedly associated with emotion regulation ([64] for review). In the current case, neurons in these areas might be involved in exerting control over an affective response that might have been automatically triggered by the sight of a highly aversive situation. The requirement of affective control might be conveyed by neurons in medial and lateral OFC, providing crucial information about the actual emotional valence of the stimuli [65]. The importance of the OFC in the regulation of empathic responses is also documented by the fMRI results of [9], [24] mentioned above, which both required regulation of one's own emotional evaluation of an aversive situation.

While our results are well in line with our hypotheses concerning the sources of emotion appraisal, a more complex picture emerges for their sites. Top-down control did not affect early perceptual processing. Even lowering thresholds to liberal levels did not reveal any significant clusters in primary or secondary visual cortex. Such an early interference might have been expected though, given the mixed blocked/event-related design which enabled participants to use anticipatory regulation. Interestingly, a previous study of our lab [24] also did not detect any modulation in visual-perceptive areas during different cognitive appraisals of painful facial expressions.

The somatosensory cortex is another potential site of activation modulation. Based upon the results of experiment I, we hypothesized that the primary somatosensory cortex is involved in matching the empathizer's bodily sensations with those of the target and that this matching allows a distinction between the painful vs. non-painful response of the anesthetized hand. The trend towards significance provides some evidence for this interpretation, but future studies are required to assess the effect size and the robustness of this finding. In addition, future studies might want to use a separate localizer task in which the first-hand somatosensory representations of touch or pain are localized in each subject.

As for activation reductions in the affective sharing network, the whole-brain and the ROI analyses suggest that all conditions triggered similar neural responses in the anterior insula and in MCC/ACC. In all ROIs a pronounced and more or less canonical hemodynamic response was observed. Signal time-courses and amplitudes were hardly distinguishable across stimulus conditions. These time-courses suggest that an automatic response was triggered by the presentation of an aversive and putatively noxious stimulus, resulting in the mobilization of withdrawal-related neural response. Note that the anticipation of a potentially painful stimulus alone is sufficient to activate large aspects of the pain matrix [54], [55], [66].

While all ROIs showed significant signal changes, it should also be noted that amplitudes in the right anterior insula and in rostral aMCC were lower during the perception of biopsies than during the perception of injections. This signal reduction might reflect the cognitively mediated down-regulation of the automatic affective response. The specific modulation of the right insula lends support to the hypothesized higher sensitivity of right as opposed to left anterior insula to various subjective feelings such as anger, coolness, disgust, trustworthiness or sexual arousal ([67], for review). The observed lateralization is also in line with the idea that the right anterior insular/opercular cortex plays a specific role in interoceptive awareness and the representation of visceral responses associated with emotional situations . These ‘gut feelings’ are thought to provide a substrate for subjective feeling states that are accessible to conscious awareness and hence cognitive appraisal [35], [67]. Such a viewpoint on emotions stresses the importance of embodied processes and their perception, representation, and appraisal by the organism (e.g., [56], [68].

In addition to the initial amplitude reduction in raMCC, a second signal increase was detected for the actually painful stimuli. This finding might reflect a second evaluation of the triggered pain – in some sense a closer or second look at the actual aversiveness of the stimulation, which is only required for the injection but not for the trials with the numbed hand. The idea about a second or late cognitive appraisal of the painful consequences receives support from recent electroencephalographic studies demonstrating late responses during the observation of painful situations in others [18], [69]. Note also that albeit we used a rapid event-related design the occurrence of a second peak during intensity ratings only cannot be explained by subsequent trials as interstimulus intervals and stimulus order were randomized and counterbalanced. However, future studies with interstimulus intervals allowing for a full return of the hemodynamic response to baseline levels are required to unequivocally exclude this potential confound. On a methodological level, the ROI analyses demonstrate the usefulness of fMRI analyses that are free of assumptions about the signal time-course and enabling to track changes deviating from the standard hemodynamic response shape.

Finally, the correlation analyses corroborate and refine the findings of the contrast analyses. The correlation of activation differences between numbed and non-numbed stimuli with the pain ratings basically identified the “classical” network detected in studies on empathy for pain. The correlation analyses also supports the interpretation that right anterior insula is more sensitive to affective variations, and that precuneus and TPJ play a specific role in distinguishing between the sensory painful and non-painful events. In addition, they indicate that areas in ipsi- and contralateral pre- and postcentral gyrus might play a more important role in this distinction than the contrast analyses alone suggest.

### Conclusion

Our study demonstrates that the perception of pain in others results in the activation of almost the entire pain matrix - including its sensory-discriminative component. Moreover, both the sensory-discriminative and the affective-motivational component is modulated by the context in which pain has occurred, and by the consequences the observer is focusing on. Interestingly, even knowing in advance that the target is not in pain triggers a similar response as when the target actually perceives pain. This is suggestive of an automatic reaction that might not be specific to pain as such but to being exposed to aversive and potentially threatening situations in general. It also casts some doubts on simulation accounts of empathy, which claim that the commonalities in the anterior insula and anterior cingulate cortex indicate the actual emotion sharing between observer and target (see also [70]). In the case of the biopsies on the numbed hand, however, no affect has to be shared and yet insular and cingulate cortices are clearly activated. This initial response might be down-regulated by cognitive mechanism of top-down control. It should be acknowledged that the low temporal resolution of hemodynamic responses might not yield precise enough information about when and how this top-down modulation affects neural activities in the pain matrix. To address this methodological limitation, we are now replicating this paradigm using event-related potential measures. In addition, future studies might want to use online interactions between observer and target to increase the ecological validity of the design. Summing up, our findings shed further light on the crucial role of cognitive processing for the experience of empathy. They demonstrate that in order to achieve a full understanding of this complex phenomenon, we need to frame it as a complex interplay between automatic and bottom-up driven and controlled top-down processes that result in a joint but highly malleable and individual experience.

## Materials and Methods

### General design

Forty-four healthy individuals were recruited for this study, which consisted of (1) a behavioral experiment and (2) two subsequent fMRI experiments (evaluative focus and appraisal). The goal of the behavioral experiment was to establish and validate the stimuli and procedures used in the fMRI experiments. Individuals who participated in the behavioral study were not involved in the fMRI experiments to avoid learning and habituation effects. A number of behavioral and dispositional measures were also taken from the fMRI participants.

### Participants

Twenty-three right-handed volunteers (19 females, mean = 27.69 years, S.D. = 3.5) participated in the behavioral experiment designed for stimulus selection and validation. Eighteen different right-handed healthy volunteers (9 females) aged between 19 and 35 years (mean = 23.67 years, S.D. = 3.99) participated in the two fMRI experiments (role of evaluative focus; role of appraisal). All participants gave informed written consent and were paid for their participation. No subject had any history of neurological, psychiatric or major medical disorder. The study was approved by the local Ethics Committee, and conducted in accordance with the Declaration of Helsinki.

### Behavioral experiment

The purpose of the behavioral experiment was to investigate whether participants are able to assess the sensory and the affective consequences resulting from needle injections into another person's hand. Participants watched photographs showing injections into different parts of the hands off different targets. After each photograph, they rated – in separate trials - the intensity or the unpleasantness of pain caused by these injections on a VAS scale. Another goal of the behavioral experiment was to identify those situations which triggered the strongest differences between the two types of rating, and to assess potential habituation effects due to the repeated exposure to similar stimuli.

#### Materials

A series of 123 digital color photographs showing pain inflicted by needle injections into the left hand of three male and three female targets was used (Figure 1). Hands were placed on a blue uniform background to suggest that pictures had been taken in a medical environment. The needle was injected into different parts of hands and fingers (e.g., close to the nail bed or next to one of the joints) to obtain variation in perceived pain intensity and unpleasantness. None of the photographs showed bleeding, but all of them showed compression and displacement of the skin around the punctured area. In addition, 42 photographs depicting neutral non-painful situations were taken. For those stimuli, the needle was covered with a black plastic cap and placed next to one the fingers. The spatial locations of this protected needle were roughly matched with those of the painful stimuli (Figure 1).

#### Procedure

After each stimulus, participants had to rate, from their perspective as viewers, pain intensity or unpleasantness using a VAS. For pain intensity ratings, the question “How much does it hurt?” had to be answered by moving a cursor between the extreme values “no pain” and “worst imaginable pain”. In the case of pain unpleasantness ratings, the question was “How unpleasant is it?”, and the VAS ranged from “not unpleasant” to “extremely unpleasant”. The difference between the sensory and the affective consequences of the painful stimulations were explained using standardized written instructions and using a number of practice trials. Stimuli were presented in eight blocks containing 41 randomly interspersed painful or non-painful stimuli each. Breaks could be taken between blocks. Prior to each block, an instruction screen informed participants whether they had to evaluate pain intensity or pain unpleasantness. The Presentation software (Neurobehavioural Systems^TM^, Albany, CA, USA) and a laptop with a TFT screen were used for stimulus presentation and response collection. The screen position of the cursor on the VAS was converted to values ranging from 0 to 100. The time to respond was not restricted, and the VAS slider was moved using the left and right arrows on the laptop keyboard. All participants used their right dominant hand to enter responses.

### Functional MRI experiments

#### Behavioral data and dispositional measures

A number of dispositional measures and behavioral data were collected in and outside of the MRI scanner to assess participants' responses to the different stimuli and conditions, as well as to assess the correlation between hemodynamic responses on the one hand and behavioral data and individual differences in empathic concern, personal distress and other variables on the other hand. In the scanner, ratings of the intensity and the unpleasantness of the inflicted pain were collected using the VAS used in the behavioral experiment. Mean VAS values of conditions were analyzed using a 2×2 repeated measures analysis of variance (ANOVA). Behavioral data and dispositional measures (including pre-test data) were analyzed using SPSS 12.0.1 (SPSS Inc., Chicago, IL, USA), and the significance threshold was set to *P* = 0.05.

Three questionnaires were filled in by the participants: the Interpersonal Reactivity Index [25], the Emotional Contagion Scale [26], and the Sensitivity to Pain Questionnaire [27]. The Interpersonal Reactivity Index (IRI) is probably the most widely used self-report measure of dispositional empathy. Its four subscales (Empathic Concern, Perspective Taking, Fantasy Scale and Personal Distress) assess different aspects of interpersonal affective responses. The Emotional Contagion Scale (ECS) assesses the susceptibility to other's emotions from afferent feedback generated by mimicry, using questions such as “I clench my jaws and my shoulders get tight when I see the angry faces on the news”. Such bodily reactions were expected during the viewing and evaluating of photographs showing painful situations. The Sensitivity to Pain Questionnaire (SPQ) assesses the participants' sensitivity to pain by asking them to assess the amount of stimulus-induced pain they would experience in fifteen painful and fifteen non-painful situations. Based on signal detection theory, a discrimination score (P(A)) and a response bias score (B) is calculated. P(A) indicates the extent to which participants are able to differentiate between painful and non-painful situations while the response bias indicates the degree to which the situations are considered as painful. We explored whether these two scores would modulate signal changes in areas of the pain matrix.

### Experimental design and procedures

In the first fMRI experiment (role of evaluative focus) we investigated how attending to the sensory or the affective consequences of painful stimulation affects regional hemodynamic responses. Participants watched photographs of targets undergoing painful and non-painful surgical procedures and rated pain intensity or pain unpleasantness. A subset of the stimuli used in the behavioral experiment was used for fMRI experiment I. In the second fMRI experiment (role of appraisal), different stimuli and targets were used. Participants were told to watch photographs taken at a local hospital, displaying two successive steps of a surgical procedure performed on the hand. One set of photographs showed the numbing of the hand using novocaine while a second set displayed a tissue biopsy performed on the numbed hand. For both experiments, a mixed blocked/event-related presentation mode and a 2×2 factorial design were implemented.

#### Experiment I (role of evaluative focus)

The two experimental factors were the stimulus type (*painful vs. non-painful*) and the evaluative focus (pain *intensity vs.* pain *unpleasantness*). A total number of 144 event-related trials were presented in two functional imaging runs (36 trials per condition). Each run contained one block in which participants had to rate the intensity or the unpleasantness of pain. The same standardized written instruction as in the behavioral experiment was used to explain the difference between these two aspects of the pain response. Several practice trials were performed before entering the scanner to ensure appropriate understanding of the instructions and experimental procedures. Each block was preceded by an instruction screen followed by the presentation of 36 painful or non-painful situations in a randomized sequence. A trial consisted of the presentation of a painful or non-painful situation (see above, behavioral experiment) for a duration of 1 s, followed by a white fixation cross or a response screen displaying the VAS (which was replaced by a fixation cross upon responding). Actual pain ratings were requested for 10 randomly selected trials out of the 36 trials presented in each condition and block. The time limit to enter a response was set to 5 s. The inter-stimuli interval was jittered (mean = 3.5 s, minimum/maximum = 2/5.8 s) to reduce stimulus predictability and to allow efficient event-related signal estimation [71]. Each stimulus was presented twice – once during the intensity rating condition and once during the unpleasantness rating condition – but stimuli were not repeated within the same run. The order of blocks was counterbalanced across participants. The results from the behavioral experiment were used to select optimal stimuli for the fMRI experiment. Out of the 123 available painful stimuli, those showing the strongest difference between intensity and unpleasantness ratings were selected. Additional selection criteria were that stimuli should show high intensity and unpleasantness ratings, and small interindividual variation in ratings.

#### Experiment II (role of appraisal)

120 event-related trials were presented in two functional imaging runs (30 trials per condition). The experimental factors were painful injections *vs.* non-painful injections (*non-numbed vs. numbed*) and the rating condition (pain *intensity vs.* pain *unpleasantness*). Stimuli for the non-numbed hand were shot with the same metallic syringe that had been used in the behavioral experiment and fMRI experiment I. For the numbed-hand stimuli, a white plastic syringe (with the same type and size of needle mounted on it as for the painful injections) was used to allow for easier discrimination. Also, the background was green in order to emphasize the difference from experiment I, and targets differed (Figure 2). According to the explicit verbal and written instructions, the painful novocaine injections and the subsequent biopsies on the numbed hand differed in one crucial aspect. While the numbing of the hand resulted in complete elimination of the somatosensation of pain for the target, the targets experienced unpleasantness and discomfort triggered by the surgical procedure (in the same way as dental work on anesthetized teeth might not be painful, but still unpleasant). Each run contained 12 blocks, with each block consisting of either five numbed hand or five injection trials. Before each block, participants were instructed by a screen insert which type of stimuli they would see, and which aspect of the pain response they were supposed to evaluate (intensity *vs.* unpleasantness). A trial consisted of the presentation of an injection or numbed-hand stimulus, for a duration of 1.7 s, followed by a fixation cross or a response screen displaying the VAS. Actual pain ratings were requested in 12 trials randomly selected out of the 30 trials for each condition. The time limit to enter a response was set to 5 s. Between trials, a white fixation cross was presented on black background, and the interstimulus interval was jittered (mean = 3.5 s, minimum/maximum = 2.2/5.8 s). The order of blocks was counterbalanced across participants.

### Function MRI data acquisition and analysis

MRI data were acquired on a 3 Tesla head-only Siemens Magnetom Allegra System equipped with a standard quadrature head coil. Changes in blood-oxygenation-level-dependent (BOLD) T2*-weighted MR signal were measured using a single-shot echoplanar imaging (EPI) sequence (repetition time TR = 1810 ms, echo time TE = 30 ms, flip angle = 80°, 30 axial slices/volume with 4.5 mm slice thickness, no gap, in-plane resolution = 3.28×3.28 mm^2^, 64×64 matrix, FOV 210×210 mm^2^). Each run was preceded by several dummy scans ensuring steady state magnetization conditions. A total of 500 EPI volumes was acquired in the two separate runs for experiment I, and 610 volumes were collected in the two runs performed for experiment II. Experiment II was always performed after experiment I. The reason for this was to avoid potential confusion and carry-over effects from the numbed hand stimuli to the non-painful stimuli from experiment I. An ascending interleaved sequence with no temporal gap between consecutive image acquisitions was used for all functional scans. The influence of in-plane susceptibility gradients in orbitofrontal regions was reduced by orienting image slices according to recommendations by [72].

Stimulus presentation and response collection were performed using the Presentation software (Neurobehavioural Systems^TM^, Albany, CA, USA). Visual stimuli were presented using a back-projection system, and a button box consisting of five buttons recorded the responses of subjects (entered using the dominant right hand).

Image processing was carried out using SPM2 (Wellcome Department of Imaging Neuroscience, London, UK), implemented in MATLAB 6.5 (Mathworks Inc., Sherborn, MA, USA). Preprocessing included slice-timing correction (with the reference slice set to the slice containing the superior-inferior center of the insula), correction for head motion (realignment to mean image volume, using the unwarp and realign function of SPM2 to account for susceptibility-movement interactions in orbitofrontal regions), normalization to the EPI template provided in SPM2, and smoothing using a 6 mm FWHM isotropic Gaussian kernel. Event-related responses were assessed by setting up fixed effects general linear models (GLM) for each subject. Regressors of interest modeling the experimental conditions, the instruction display and the evaluation epochs were set up and convolved with the standard canonical hemodynamic response function. Fixed effects models incorporated a high-pass filter with a frequency cut-off at 128 s. Following model estimation, contrasts were calculated for each subject to assess differences between conditions. In addition, signal changes in relationship to the inherently modeled baseline (i.e., fixation) were assessed. The resulting first-level contrast images were entered into second-level random effects analyses to assess differences between conditions with population inference.

Activity differences between the presentation of *physically* distinct stimuli (*painful vs. non-painful* photographs, and photographs *vs.* fixation) were interpreted using a voxel-level threshold of *P* = 0.01 and a spatial extent threshold of *k* = 10, corrected for multiple comparisons across the whole volume using the false discovery rate (FDR) approach [73]. The more subtle differences in signal strength between conditions that differed *psychologically* (e.g., *intensity* vs. *unpleasantness* ratings, *painful injection* vs. *numbed injection*) were thresholded using a more liberal threshold of *P* = 0.001 (uncorrected for multiple comparisons) and an extent criterion of *k* = 5. The choice of these thresholds was based upon exploratory data analyses and upon effect size considerations derived from similar experiments of our own and other groups [3], [4], [8], [9], [24]. In addition, the threshold was lowered to *P* = 0.005 (uncorrected), *k* = 5, for *a priori* defined regions involved in the perception of pain and in emotion regulation in order to assess whether they showed activation below threshold. Significant clusters were anatomically labeled using structural neuroanatomy information and probabilistic cytoarchitectonic maps provided in the Anatomy Toolbox (version 1.4; [31] and the Anatomic Automatic Labeling toolbox (AAL; [74]). For brain regions not covered by these toolboxes, the brain atlas of [75] was used. Nomenclature for activations in cingulate cortex is based on a recent review of cingulate anatomy and function [32].

For both fMRI experiments, target analyses evaluated the interactions between the two experimental factors. For *experiment I*, the interaction contrast *Intensity (Pain>No Pain)>Unpleasantness (Pain>No Pain)* assessed which brain areas responded more to evaluating the sensory aspects of the stimulation – controlling for the generalized response to the non-painful stimuli. The reverse interaction identified clusters indicating stronger activation related to affective evaluations, again controlling for the generalized response to the depiction of the hand and an aversive object.

For *experiment II*, the same analysis approach was used. Here, the interaction term assessed activation modulations in areas involved in intensity ratings of injections to the numbed and non-numbed hand and contrasted it with the expected absence of such differences for the unpleasantness ratings. In addition, a direct comparison between numbed and painful injections for intensity rating trials only explored additional potential differences not detected by the interaction contrast.

Complementary to the whole-brain analyses, region-of-interest analyses were performed using the MarsBaR toolbox, v0.38 (http://www.sourceforge.net/projects/marsbar). These analyses compared event-related hemodynamic responses in *a priori* defined functional ROIs. The average signal of all voxels in a certain ROI was extracted per TR in a peristimulus epoch of 15 TRs (i.e., about 27 s). For fMRI experiment II individual ROIs of activations coding the affective-motivational consequences of painful stimulation were defined guided by a meta-analysis of insular and cingulate cortex activation during the perception of pain in self and others [34]. Two ROIs in left and right anterior insula and three ROIs in cingulate cortex were defined. ROIs in cingulate cortex were located in ventral posterior MCC (vpMCC), in dorsal aMCC (daMCC), and in rostral aMCC (raMCC; see Table S2). Individual functional ROIs were delineated by determining the conjunction (∩) of the activation map (*P* = 0.05, uncorrected, contrast All_Painful>All_Non-painful from fMRI experiment I, with All referring to activation being pooled across both rating conditions) with a boundary box with dimensions 10×10×10 mm drawn around the peak coordinate. In addition, to scrutinize whether cognitive appraisal modulates somatosensory representations, a combined functional-anatomical ROI from contralateral primary somatosensory activation was determined for each subject. The boundaries of this ROI consisted of the conjunction of supra-threshold activation in contralateral (right) postcentral gyrus with the cytoarchitectonic delineation of Area 2 provided in the Anatomy toolbox. The reason for this different approach was that activation in contralateral somatosensory cortex was less focal than for the other ROIs, showed more variability across subjects, and that a clear-cut cytoarchitectonic and anatomical delineation of this area was available. Area 2 (instead of the other somatosensory areas) was chosen because it was the only area in postcentral gyrus showing significant activation in the random effects grand mean activation map.

Statistical analysis of ROI data consisted of computing planned comparisons on signal peaks (which usually occurred around the third to fourth TR post-stimulus, i.e. about 5–7 s post stimulus). The planned comparisons followed the same analysis approach as the whole brain analyses: First, we tested the interaction term (Intensity: numbed vs. not-numbed≠Unpleasantness: numbed vs. not-numbed). Then, we directly compared numbed and not-numbed for the intensity trials only. In all cases, violations of the sphericity-assumption for these comparisons were accounted for by using specific error-variances [76].

In order to assess the relationship between behavioral data and brain activation, random effects correlation analyses were performed. Scores of the Empathic Concern scale of the IRI, the ECS, and values P(A) and B of the SPQ were correlated with individual contrast maps. In accordance with other studies assessing brain-behavior relationships, a rather liberal significance threshold of *P* = 0.001 (uncorrected) and *k = *5 was selected for these analyses.

## Supporting Information

Figure S1 Significant clusters revealed by the interaction contrast (Intensity: Injection>No injection)>(Unpleasant: Injection>No injection ) from fMRI experiment I. Threshold P = 0.001 (uncorrected), k = 5.
(1.96 MB TIF)

Figure S2 Significant clusters revealed by the contrast numbed>injection (for intensity rating trials only). Threshold P = 0.005 (uncorrected), k = 5.
(2.04 MB TIF)

Figure S3 Significant clusters revealed by the contrast injection>numbed (for intensity rating trials only). Threshold P = 0.001 (uncorrected), k = 5.
(1.99 MB TIF)

Table S1 Mean scores and standard deviations for the dispositional measures for the sample investigated.
(0.03 MB RTF)

Table S2 Peak coordinates of clusters identified for ROIs in anterior insula (AI), dorso-medial anterior cingulate cortex (daMCC), rostral aMCC (raMCC), and ventral aMCC (vaMCC).
(0.06 MB DOC)

Table S3 Significant correlations of hemodynamic responses from fMRI experiment I with emotion contagion score, empathic concern score, scores of the situational pain questionnaire, and with pain intensity and unpleasantness ratings.
(0.13 MB DOC)

Table S4 Significant correlations of hemodynamic responses from fMRI experiment II with emotion contagion score, perspective taking score and rating score differences.
(0.09 MB DOC)
