# Supplementary figures and images for: What Are You Feeling? Using Functional Magnetic Resonance Imaging to Assess the Modulation of Sensory and Affective Responses during Empathy for Pain
Source: PLoS One. 2007 Dec 12;2(12):e1292. doi: 10.1371/journal.pone.0001292 (PMC2144768; doi:10.1371/journal.pone.0001292)

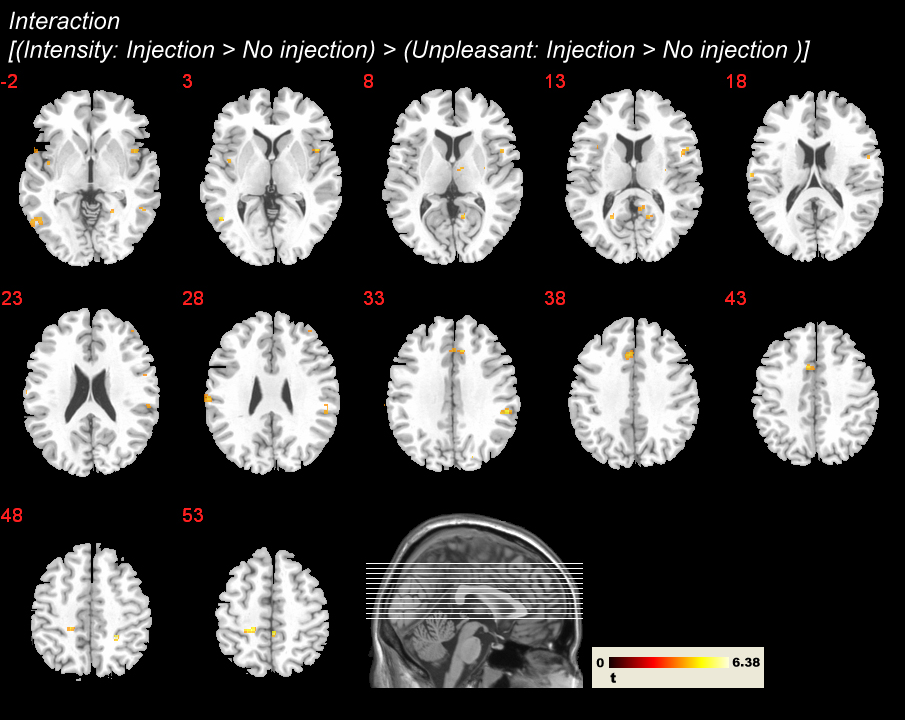

Supplement: Figure S1 — Significant clusters revealed by the interaction contrast (Intensity: Injection>No injection)>(Unpleasant: Injection>No injection ) from fMRI experiment I. Threshold P = 0.001 (uncorrected), k = 5. (1.96 MB TIF) [file pone.0001292.s001.tif]

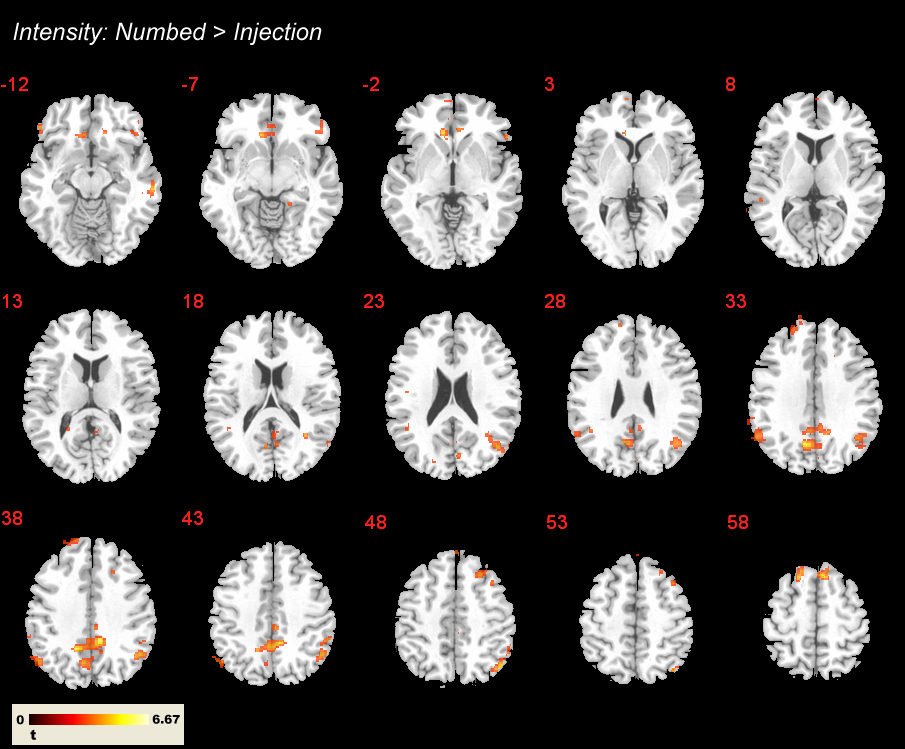

Supplement: Figure S2 — Significant clusters revealed by the contrast numbed>injection (for intensity rating trials only). Threshold P = 0.005 (uncorrected), k = 5. (2.04 MB TIF) [file pone.0001292.s002.tif]

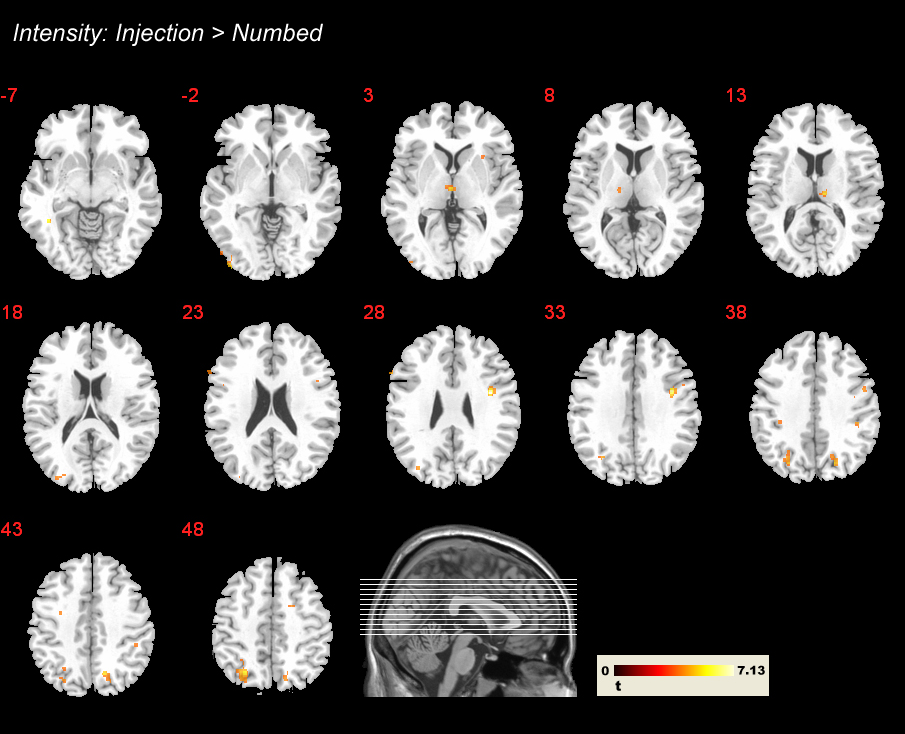

Supplement: Figure S3 — Significant clusters revealed by the contrast injection>numbed (for intensity rating trials only). Threshold P = 0.001 (uncorrected), k = 5. (1.99 MB TIF) [file pone.0001292.s003.tif]
