# Supplementary material for: What Are You Feeling? Using Functional Magnetic Resonance Imaging to Assess the Modulation of Sensory and Affective Responses during Empathy for Pain
Source: PLoS One. 2007 Dec 12;2(12):e1292. doi: 10.1371/journal.pone.0001292 (PMC2144768; doi:10.1371/journal.pone.0001292)
Supplement: Table S2 — Peak coordinates of clusters identified for ROIs in anterior insula (AI), dorso-medial anterior cingulate cortex (daMCC), rostral aMCC (raMCC), and ventral aMCC (vaMCC). (0.06 MB DOC) [file pone.0001292.s005.doc]

|  | *Left AI* | | | *Right AI* | | | *dMCC* | | | *rvMCC* | | | *vACC* | | |
| --- | --- | --- | --- | --- | --- | --- | --- | --- | --- | --- | --- | --- | --- | --- | --- |
|  | *x* | *y* | *z* | *x* | *y* | *z* | *x* | *y* | *z* | *x* | *y* | *z* | *x* | *y* | *z* |
| subject |  |  |  |  |  |  |  |  |  |  |  |  |  |  |  |
| 01 | *-32* | *22* | *4* | *30* | *18* | *6* | *4* | *14* | *52* | *8* | *32* | *36* | *0* | *4* | *34* |
| 02 | *-34* | *20* | *-4* | *36* | *24* | *-4* | *2* | *16* | *42* | *8* | *28* | *36* | *0* | *2* | *30* |
| 03 | *-28* | *24* | *2* | *32* | *22* | *2* | *-6* | *18* | *48* | *-4* | *28* | *28* | *6* | *8* | *28* |
| 04 | *-34* | *26* | *0* | *40* | *20* | *0* | *0* | *20* | *44* | *-2* | *30* | *32* | *2* | *2* | *38* |
| 05 | *-28* | *20* | *2* | *34* | *24* | *-10* | *6* | *24* | *44* | *-4* | *32* | *36* | *-2* | *4* | *38* |
| 06 | *-36* | *18* | *4* | *28* | *26* | *6* | *-6* | *12* | *46* | *-6* | *30* | *32* | *2* | *-4* | *32* |
| 07 | ∅ | | | *32* | *30* | *0* | *4* | *18* | *50* | ∅ | | | *-2* | *4* | *34* |
| 08 | *-36* | *26* | *-8* | *32* | *14* | *4* | *-2* | *24* | *44* | ∅ | | | *6* | *6* | *36* |
| 09 | *-38* | *26* | *-2* | *34* | *32* | *-2* | *0* | *16* | *54* | *2* | *32* | *36* | *4* | *-2* | *36* |
| 10 | *-36* | *20* | *-6* | *34* | *28* | *0* | *6* | *8* | *52* | ∅ | | | ∅ | | |
| 11 | *-44* | *16* | *2* | *38* | *12* | *-8* | *-4* | *6* | *54* | ∅ | | | *-12* | *28* | *28* |
| 12 | *-36* | *30* | *0* | *34* | *18* | *2* | *4* | *30* | *44* | *-8* | *40* | *24* | *2* | *10* | *32* |
| 13 | *-30* | *24* | *-10* | *36* | *26* | *-6* | *0* | *18* | *50* | *8* | *32* | *36* | *2* | *24* | *22* |
| 14 | *-36* | *22* | *-2* | *34* | *26* | *0* | *2* | *8* | *48* | *-2* | *18* | *38* | *0* | *-4* | *28* |
| 15 | *-36* | *26* | *6* | *28* | *18* | *8* | *8* | *14* | *46* | *-4* | *18* | *38* | *2* | *2* | *30* |
| 16 | *-34* | *26* | *-4* | *32* | *22* | *-2* | *2* | *16* | *50* | *-8* | *36* | *32* | *-4* | *0* | *34* |
| 18 | *-36* | *20* | *-2* | *32* | *20* | *0* | *2* | *24* | *44* | *4* | *30* | *34* | *-2* | *6* | *32* |

∅ = no supra-threshold voxels; The mean coordinates of these ROIs were as follows:

Left AI: -34.63/22.88/-1.13, right AI: 33.29/22.35/-0.24, daMCC: 1.29/16.82/47.76, raMCC: -0.62/29.69/33.69, vaMCC: 0.25/5.63/32
