# Supplementary material for: What Are You Feeling? Using Functional Magnetic Resonance Imaging to Assess the Modulation of Sensory and Affective Responses during Empathy for Pain
Source: PLoS One. 2007 Dec 12;2(12):e1292. doi: 10.1371/journal.pone.0001292 (PMC2144768; doi:10.1371/journal.pone.0001292)
Supplement: Table S3 — Significant correlations of hemodynamic responses from fMRI experiment I with emotion contagion score, empathic concern score, scores of the situational pain questionnaire, and with pain intensity and unpleasantness ratings. (0.13 MB DOC) [file pone.0001292.s006.doc]

|  | *L/R/M* | *k* | *x* | *y* | *z* | *cc* |
| --- | --- | --- | --- | --- | --- | --- |
| **Emotion Contagion Score ↔ All_Painful** | | | | | | |
| Precuneus | R | 43 | 16 | -68 | 44 | 0.84 |
| Precuneus | L | 7 | -18 | -50 | 10 | 0.81 |
| Supramarginal Gyrus | R | 77 | 58 | -38 | 32 | 0.83 |
| Angular Gyrus | L | 22 | -48 | -50 | 34 | 0.80 |
| Inferior Parietal Cortex | R | 25 | 26 | -46 | 48 | 0.73 |
| x Angular Gyrus | R |  | 28 | -48 | 40 | 0.72 |
| Cuneus | M | 11 | -8 | -70 | 24 | 0.73 |
| Lingual Gyrus | M | 6 | 8 | -36 | 2 | 0.74 |
| Fusiform Gyrus | L | 5 | -40 | -56 | -10 | 0.73 |
| Middle Occipital Gyrus | L | 28 | -42 | -70 | 16 | 0.81 |
| Anterior Insula | R | 17 | 48 | -8 | 4 | 0.78 |
| Anterior Insula | R | 11 | 34 | 22 | -6 | 0.73 |
| Anterior Cingulate Cortex | M | 8 | 2 | 38 | 10 | 0.73 |
| Anterior Cingulate Cortex | M | 8 | -8 | 34 | 30 | 0.70 |
| Precentral Gyrus | R | 30 | 42 | 6 | 44 | 0.77 |
| x Precentral Gyrus | R |  | 52 | 2 | 48 | 0.73 |
| Superior Frontal Gyrus | L | 5 | -30 | 58 | 0 | 0.73 |
| Middle Frontal Gyrus | R | 6 | 32 | 56 | 26 | 0.72 |
| Inferior/Orbitofrontal Cortex | L | 11 | -36 | 24 | -10 | 0.76 |
| Medial Orbitofrontal Cortex | M | 12 | -2 | 44 | -20 | -0.78 |
| **Empathic Concern ↔ All_Painful > All_Non-painful** | | | | | | |
| Precentral Gyrus | R | 21 | 40 | 0 | 28 | 0.78 |
| Middle Frontal Gyrus | R | 6 | -24 | 10 | 48 | 0.71 |
| Middle Frontal Gyrus | R | 12 | 46 | 26 | 32 | 0.75 |
| Inferior Frontal Gyrus | L | 25 | -56 | 28 | 16 | 0.81 |
| Inferior Frontal/Orbitofrontal Cortex | L | 19 | -26 | 28 | -22 | 0.77 |
| x Inferior Frontal/Orbitofrontal Cortex | L |  | -28 | 30 | -14 | 0.75 |
| Middle Occipital Gyrus | L | 9 | -36 | -62 | 16 | 0.80 |
| Fusiform Gyrus | L | 8 | 32 | -42 | -20 | 0.74 |
| Fusiform Gyrus | L | 5 | -32 | -44 | -22 | 0.70 |
| Middle Occipital Gyrus | L | 15 | 38 | -78 | 36 | 0.69 |
| Precuneus | M | 7 | 8 | -66 | 46 | 0.70 |
| Inferior Parietal Cortex | L | 18 | -46 | -34 | 42 | 0.76 |
| **SPQ: P(A) ↔ All_Painful > All_Non-painful** | | | | | | |
| Fusiform Gyrus | L | 14 | -30 | -60 | -6 | -0.80 |
| Middle Occipital Gyrus | R | 17 | 24 | -88 | 6 | -0.79 |
| Perigenual ACC | R | 17 | 12 | 38 | -6 | -0.77 |
| Anterior Insula/Fronto-insular Cortex | L | 6 | -24 | 22 | -12 | -0.73 |
| **SPQ: B ↔ All_Painful > All_Non-painful** | | | | | | |
| Subcallosal ACC/medial OFC | M | 27 | -8 | 26 | -12 | -0.84 |
| Anterior Insula/Fronto-insular Cortex | L | 5 | -24 | 22 | -10 | -0.73 |
| Fusiform Gyrus | R | 5 | 22 | -40 | -14 | -0.71 |
| **Intensity Rating ↔ Intensity_Painful** | | | | | | |
| Inferior Temporal Gyrus | R | 225 | 56 | -56 | -4 | 0.78 |
| Inferior Temporal Gyrus | L | 26 | -50 | -66 | -8 | 0.72 |
| Superior Temporal Gyrus | R | 19 | 52 | -40 | 18 | 0.72 |
| Inferior Frontal Gyrus | L | 15 | -44 | 22 | 6 | 0.68 |
| Inferior Frontal Gyrus (Area 45) | L | 14 | -52 | 34 | 14 | *0.67 |
| Inferior Frontal Gyrus (Area 45) | R | 23 | 54 | 28 | 26 | 0.68 |
| Middle Frontal Gyrus | L | 23 | -24 | 16 | 58 | 0.73 |
| Posterior Cingulate Cortex | M | 19 | 6 | -36 | 40 | 0.75 |
| Middle Frontal Gyrus/Precentral Gyrus | R | 8 | 32 | 0 | 54 | *0.62 |
| **Unpleasantness Rating ↔ Unpleasantness_Painful** | | | | | | |
| Inferior Temporal Gyrus | R | 117 | 46 | -60 | -10 | 0.77 |
| Inferior Temporal Gyrus (Area 45) | L | 86 | -42 | 32 | 16 | 0.77 |
| Inferior Temporal Gyrus | L | 16 | -50 | -42 | -16 | 0.73 |
| Inferior Temporal Gyrus | L | 40 | -50 | -64 | -8 | 0.68 |
| Middle Temporal Gyrus | R | 15 | 56 | -70 | 0 | 0.70 |
| Middle Temporal Gyrus | L | 19 | -48 | -56 | 4 | 0.70 |
| Inferior Occipital Gyrus | R | 27 | 30 | -82 | -14 | 0.74 |
| Middle Occipital Gyrus | R | 19 | 44 | -76 | 14 | 0.73 |
| Lingual Gyrus | L | 25 | -16 | -62 | -4 | 0.69 |
| Fusiform Gyrus | L | 15 | -42 | -82 | -14 | 0.69 |
| Dorsal Middle Insula | R | 13 | 40 | 0 | 10 | 0.70 |
| Postcentral/Supramarginal Gyrus  (Areas OP4/1) | L | 10 | -66 | -12 | 24 | 0.69 |
| Supramarginal Gyrus (Areas OP4/1) | R | 43 | 62 | -20 | 30 | 0.69 |
| Cerebellum | L | 9 | -38 | -48 | -30 | 0.73 |
| Middle Frontal Gyrus | L | 31 | -18 | 40 | 26 | 0.74 |
| Superior Parietal Lobe | L | 21 | -22 | -70 | 52 | 0.69 |

Legend: Voxel-level threshold *P* = 0.001 (uncorrected), cluster size threshold *k* = 5, * *P* = 0.005 (uncorrected), *k* = 5, for regions with a priori hypotheses. Stereotactic coordinates and r-values are provided for the local voxel maxima in the respective cluster. x = sub-peaks of a cluster, L = left hemisphere, R = right hemisphere, M = medial activation, k = number of activated voxels in cluster, cc = correlation coefficient (Pearson), ACC = Anterior Cingulate Cortex, OFC = Orbitofrontal Cortex.
