# Supplementary material for: What Are You Feeling? Using Functional Magnetic Resonance Imaging to Assess the Modulation of Sensory and Affective Responses during Empathy for Pain
Source: PLoS One. 2007 Dec 12;2(12):e1292. doi: 10.1371/journal.pone.0001292 (PMC2144768; doi:10.1371/journal.pone.0001292)
Supplement: Table S4 — Significant correlations of hemodynamic responses from fMRI experiment II with emotion contagion score, perspective taking score and rating score differences. (0.09 MB DOC) [file pone.0001292.s007.doc]

|  | *L/R/M* | *k* | *x* | *y* | *z* | *cc* |
| --- | --- | --- | --- | --- | --- | --- |
| **Difference between non-numbed and numbed hand ratings for pain intensity ↔ Biopsy > Injection (intensity trials)** | | | | | | |
| Fronto-Insular Cortex | L | 68 | -34 | 12 | -12 | 0.87 |
| x Anterior Insula | L |  | -34 | 14 | 0 | 0.75 |
| Anterior Insula | R | 9 | 48 | 12 | -4 | 0.75 |
| Anterior Insula | R | 6 | 36 | 26 | -6 | 0.73 |
| Precentral Gyrus | L | 45 | -44 | -6 | 50 | 0.86 |
| Precentral Gyrus | R | 117 | 38 | -14 | 44 | 0.85 |
| Precentral Gyrus | L | 8 | -34 | -10 | 44 | 0.77 |
| Postcentral Gyrus | R | 7 | 54 | -6 | 36 | 0.78 |
| Thalamus | M | 186 | -8 | -24 | 4 | 0.82 |
| x Thalamus | M |  | -12 | -14 | 12 | 0.76 |
| Supplementary Motor Area | R | 32 | 14 | 8 | 60 | 0.82 |
| Supplementary Motor Area | L | 42 | -10 | 18 | 46 | 0.79 |
| Anterior Cingulate Cortex | M | 18 | -10 | 38 | 8 | 0.82 |
| Anterior Cingulate Cortex | M | 11 | -10 | 24 | 16 | 0.80 |
| Anterior Cingulate Cortex | M | 16 | -10 | 12 | 34 | 0.76 |
| Posterior Cingulate Cortex | M | 9 | -4 | -26 | 26 | 0.73 |
| Medial Cingulate Cortex | R | 5 | 12 | 8 | 40 | 0.74 |
| Supramarginal Gyrus | R | 15 | 64 | -46 | 24 | 0.77 |
| Precuneus | M | 6 | 10 | -44 | 44 | 0.76 |
| Precuneus | M | 5 | -8 | -56 | 28 | 0.75 |
| Precuneus | M | 29 | 2 | -76 | 40 | 0.75 |
| **Perspective Taking Score ↔ Numbed > Non-numbed (intensity trials)** | | | | | | |
| Medial Insular Cortex | L | 56 | -38 | -10 | -10 | 0.64 |
| Medial Cingulate Cortex | M | 119 | 4 | -14 | 38 | 0.85 |
| Medial Cingulate Cortex | M | 5 | 6 | 24 | 36 | 0.71 |
| Anterior Cingulate Cortex | M | 68 | -6 | 14 | 22 | 0.85 |
| Perigenual Anterior Cingulate Cortex | M | 9 | -6 | 36 | 0 | 0.76 |
| Perigenual Anterior Cingulate Cortex | M | 59 | -8 | 40 | 16 | 0.76 |
| Subcallosal Anterior Cingulate Cortex | M | 5 | 8 | 28 | -8 | 0.78 |
| Caudate Nucleus | M | 134 | 8 | 20 | -2 | 0.80 |
| Supplementary Motor Area | R | 6 | 12 | 18 | 60 | 0.80 |
| Supplementary Motor Area | R | 10 | 12 | -20 | 66 | 0.79 |
| Temporal Pole | R | 66 | 50 | 14 | -10 | 0.79 |
| x Inferior Frontal Gyrus/Operculum | R |  | 50 | 16 | 2 | 0.78 |
| x Anterior Insula | R |  | 42 | 12 | -12 | 0.64 |
| Precentral Gyrus | R | 11 | 14 | -28 | 76 | 0.76 |
| Thalamus | M | 10 | 8 | -26 | 16 | 0.75 |
| **Emotion Contagion Score ↔ Biopsy > Injection (intensity trials)** | | | | | | |
| Postcentral Gyrus | L | 16 | -40 | -8 | 32 | -0.82 |
| Post/Precentral Gryus | R | 54 | -12 | -28 | 64 | -0.81 |
| Supramarginal Gyrus | R | 12 | 62 | -38 | 36 | -0.78 |
| Angular Gyrus | L | 5 | -44 | -48 | 34 | -0.78 |
| Inferior Parietal Cortex | R | 35 | 40 | -52 | 38 | -0.74 |

Legend: Voxel-level threshold *P* = 0.001 (uncorrected), cluster size threshold *k* = 5. Stereotactic coordinates and r-values are provided for the local voxel maxima in the respective cluster. x = sub-peaks of a cluster, L = left hemisphere, R = right hemisphere, M = medial activation, k = number of activated voxels in cluster, cc = correlation coefficient (Pearson)
